# Sense of coherence is linked to post-traumatic growth after critical incidents in Austrian ambulance personnel

**DOI:** 10.1186/s12888-019-2065-z

**Published:** 2019-03-11

**Authors:** Klemens Ragger, Michaela Hiebler-Ragger, Günter Herzog, Hans-Peter Kapfhammer, Human Friedrich Unterrainer

**Affiliations:** 1 University Clinic of Psychiatry and Psychotherapeutic Medicine, Medical University of Graz, Auenbruggerplatz 31, A-8036, Graz, Austria; 2 Center for Integrative Addiction Research (Grüner Kreis Society), Rudolfsplatz 9, A-1010 Vienna, Austria; 3 Department for Religious Studies, University of Vienna, Schenkenstraße 8-10/5th floor, A-1010 Vienna, Austria

**Keywords:** Post-traumatic growth, Sense of coherence, Post-traumatic stress symptoms, Critical incidents, Ambulance personnel

## Abstract

**Background:**

Ambulance personnel, as well as other emergency services like fire-fighters or the police force, are regularly confronted with experiences of extreme psychological distress and potentially traumatizing events in the line of their daily duties. As a consequence, this occupational group is exposed to an elevated risk of developing symptoms of Post-Traumatic Stress (PTSS). Subsequently, symptoms of Post-Traumatic Stress have been observed as potentially co-occurring with Post-Traumatic Growth (PTG) in ambulance personnel as well. Therefore, in this study we hypothesized that Sense of Coherence (SOC) might play an important role as an underlying feature in enabling growth after stressful experiences in Austrian ambulance personnel.

**Methods:**

In this study, voluntary and full-time ambulance personnel (*n* = 266) of the Austrian Red Cross ambulance service completed an online survey including the Sense of Coherence Scale (SOC-29), the Post-Traumatic Growth Inventory (PTGI) and the Impact of Event Scale Revised (IES-R) for the assessment of PTSS. In line with theoretical considerations, a two-step cluster analysis limited to four clusters and further ANOVAs were conducted.

**Results:**

Four clusters were confirmed and labelled PTSS-low/PTG-low, PTSS-low/PTG-high, PTSS-high/PTG-high and PTSS-high/PTG-low. Further ANOVAs revealed substantial cluster differences in SOC, with higher SOC-levels in PTSS-high/PTG-high than in PTSS-high/PTG-low (*p* < .01), in PTSS-low/PTG-high than in PTSS-low/PTG-low (*p* < .01) and in PTSS-low/PTG-high than in PTSS-high/PTG-low (*p* < .01).

**Conclusions:**

Our findings point to a significant association between SOC and the development of PTG in ambulance personnel. Furthermore, the results suggest that growth and stress after critical incidents are independent from each other and can co-exist. Therefore, promoting SOC (e.g., meaningfulness) in ambulance personnel – e.g., through psychological interventions – might preserve and enhance psychological health after critical incidents.

## Background

Emergency service members experience an increased number of potentially traumatic events (i.e., critical incidents) in the line of duty [1–4]. According to the fifth edition of the Diagnostic and Statistical Manual of Mental Disorders (DSM-V), a traumatic event is defined as the exposure to actual or threatened death, serious injury or sexual violation through personal experience, witnessing it as it occurs to other people or through the extreme exposure to aversive details of the event [5]. This applies in particular to emergency service personnel (e.g. ambulance personnel, fire fighters, police force) because of their regular confrontation with extreme psychological distress, critical incidents or potentially traumatizing events in the line of their daily duties [2]. For instance, ambulance personnel, as well as fire-fighters or police officers, is consistently confronted with vehicle accidents, incidents involving children, Sudden Infant Death Syndrome (SIDS), severe burns, suicides and events involving mass casualties [2, 6]. Therefore, it can be assumed that there is an elevated risk for ambulance personnel to experience Post-Traumatic Stress Symptoms (PTSS) which presumably threaten their psychological well-being [7]. In line with this, there is rising evidence of noticeably higher rates of Post-Traumatic Stress Disorder (PTSD) among emergency service personnel (e.g. police, fire fighters, ambulance personnel), compared to the general population [2, 3, 8, 9]. Specifically, studies among ambulance personnel revealed elevated PTSD-rates, between 10 and 22%, while the general population showed PTSD-rates between one and 3 % [3, 9–11]. However, considering the frequent confrontation of ambulance personnel with critical incidents, these PTSD rates appear to be relatively low [12]. In search of possible reasons for this discrepancy, previous studies hypothesized that critical incidents might not only lead to negative outcome (e.g., PTSS) but could also result in positive psychological changes including Post-Traumatic Growth (PTG) [1, 2].

The most established model of PTG by Tedeschi and Calhoun [13] defines it as *“positive psychological change experienced as a result of the struggle with highly challenging life circumstances”* (p. 1) [13]. Therefore, PTG can manifest itself in different ways, including a more intense social relationship to others, an enhanced awareness of new possibilities in life, an increased perception of personal strength, a deepened meaning of spirituality and an increased appreciation of life [13, 14]. Consequently, PTG would result in a widened sense of wisdom about the world and an increased satisfaction with life [13–16]. While it can be considered both as a process and as an outcome [16], PTG mostly represents the result of intentional rumination processes intended to integrate a traumatic experience into a previous view of the world, rather than a direct outcome of the traumatic event itself [15, 17]. Therefore, an individual’s core beliefs about the meaning and working of the universe, as well as their adaption to it, play an important role in the development of PTG [18]: If an experience does not challenge an individual’s core beliefs (because they can provide a possible explanation to understand the meaning of the event) the resulting PTSS as well as the potential PTG should be relatively low [15]. However, if an individual’s core beliefs are challenged, an experience is considered traumatic. In this case, psychological help could enable more effective rumination processes and consequently support the development of PTG [15]. As this process might take some time, the development of PTG is likely to happen later on in the adjustment process and represent a precious consequence of the struggle with a highly stressful life event [13, 16]. Accordingly, PTG is seen less as a coping process and more as a process of progression beyond the pre-traumatic status quo [7]. Therefore, Tedeschi and Calhoun [13–15] see PTSS and PTG as independent constructs that can also be present at the same time: In the aftermath of a traumatic event a person can experience growth out of the effort exerted in surviving the event. However, the memory of the traumatic event itself may still be distressing [13]. In accordance to this research, efforts were put into the examination of both characteristics: PTSS as well as PTG [2].

Tedeschi and Calhoun [13] differentiated PTG from related constructs, including the salutogenetic Sense of Coherence (SOC) by Antonovsky [19]. They assumed that both PTG and SOC represent personality traits which enable people to deal adequately with stressful life events [13]. Based on Antonovsky’s definition [19], SOC is a global attitude of confidence that allows a person to understand why a stressful life event occurred (comprehensibility), to manage it on their own or through the help of others (manageability) and to find a deeper meaning in it (meaningfulness) [19–21]. Therefore, people with a high SOC are more capable in handling a stressful life event, because of their ability to comprehend the event, to manage it and to find a deeper meaning in it. Furthermore, they seem to be more capable in choosing functional coping strategies [21]. In comparison, PTG relates to psychological changes beyond not being psychologically harmed by a stressful life event [13]. In fact, PTG should lead to a process of progression beyond the pre-traumatic status quo [7, 13]. This could point to a possible relationship between SOC and PTG. According to Forstmeier et al. [22] a person who is able to find a deeper meaning (SOC) in a stressful life event could also experience PTG (i.e. personal strength) in the same process. Additionally, Znoj [23] found a positive relationship between the SOC subscale meaningfulness and PTG among samples of people with a spinal cord injury as well as in bereaved parents.

The ability of finding more meaningfulness (SOC) in a distressing life event is assumed to develop while struggling with the event itself, whereby this struggling process is seen as quit essential for PTG [13]. Therefore, ambulance personnel might be very competent in enabling SOC (e.g. meaningfulness), because of their constant struggle with traumatic events in the line of duty. In turn, this constant struggle could have a positive effect on the ability of ambulance personnel to develop PTG while adjusting to a traumatic event [13]. Thus, SOC (e.g. manageability) could play an important role for ambulance personnel to reduce their stress level and to preserve their operational ability [24], which in turn could support the development of PTG. In this context, studies found indications that ambulance personnel with professional training showed a higher SOC-level and a lower PTSS-level than non-prepared colleagues [12, 25]. Moreover, the availability of a functional peer support system seems to be connected with higher SOC-levels and lower PTSS-levels among ambulance personnel [12, 25]. Therefore, studies that investigate coping mechanisms (e.g. SOC) and consequences (e.g. PTS and PTG) of experiencing traumatic events in ambulance personnel are important for efforts to target these variables in the education and daily support of this occupational group that is regularly exposed to traumatic events in the line of duty.

While a number of studies indicate that SOC is negatively related to PTSS [12, 21, 26] and positively related to PTG [22, 27], only a few studies [12, 28] could be detected where the relevance of SOC for PTSS in emergency service personnel was examined. Furthermore, up to now no studies could be found that focused on the relevance of SOC for PTG in emergency service personnel. In this study, we therefore aimed to examine the interactions of these constructs in Austrian ambulance personnel. In accordance with the literature, we formulated three hypotheses: 1. PTSS and PTG are related to SOC; 2. PTG and PTSS can exist independently from each other; 3. SOC is associated with PTG despite the coexistence of PTSS.

## Methods

### Sample description and procedure

The data collection took place from February 9th, 2016 to May 31st, 2016. The recruitment happened over the internal e-mail platform of the Styrian Red Cross and willing participants completed all relevant questionnaires online on a SurveyMonkey® platform. For that purpose, an e-mail, including the request to participate and the link to the online questionnaire, was sent to all listed active 5660 voluntary and full-time members of the Styrian Red Cross ambulance service (5067 voluntary, 593 full-time, 1593 female, 4067 male), whereby informed consent had to be given before completing the questionnaires. In regard of their current qualification level (Emergency Medical Technician vs. Critical Care Paramedic), both voluntary and full-time members underwent the same amount of professional training to certify for active duty within the ambulance service. A total of 407 people participated in the study, although 141 people had to be excluded due to incomplete questionnaires. Only fully completed questionnaires (*n* = 266) were included in further statistical analyses. The study was conducted in agreement with the Declaration of Helsinki and approved by the Ethics Committee of the Medical University of Graz, Austria.

### Measures

#### Socio-demographics

First, participants completed a socio-demographic questionnaire that included questions about the participants’ age, sex, family status and their highest completed education level. Furthermore, participants answered various questions relating to their involvement in the Red Cross ambulance service such as their professional training status, the years of service so far and the average number plus duration of ambulance services per month.

#### Impact of event scale revised (IES-R)

Post-Traumatic Stress symptoms (PTSS) were measured by means of the 22-item German version of the Impact of Event Scale Revised (IES-R) [29, 30], which has been proven as a reliable tool for the measurement of the three major DSM-IV-TR criteria for Post-Traumatic Stress: Intrusion, Avoidance and Hyperarousal [7, 31, 32]. The IES-R is made up of these three subscales with a satisfying internal consistency (Cronbach α in brackets): Intrusion (7 items, e.g. ‘I had trouble staying asleep’; *α* = .87), Avoidance (8 items, e.g. ‘I tried not to think about it’; *α* = .84) and Hyperarousal (7 items, e.g. ‘I felt irritable and angry’; *α* = .79) [7]. The items were rated on a 4-point Likert scale (0 = “Not at all”; 1 = “Rarely”; 3 = “Sometimes”; 5 = “Often”; 1,5) and the probability for a PTSD-diagnosis was calculated by means of a regression formula (X = − 0.02*Intrusion+ 0.07*Avoidance+ 0.15*Hyperarousal-4.36) [29]. According to relevant literature a high probability for a current PTSD could be presumed if the regression formula resulted in a positive X-value. Otherwise, if the regression formula resulted in a negative X-value, a current PTSD could be presumed as unlikely [29].

#### Post-traumatic growth inventory (PTGI)

The 21-item German version of the Post-Traumatic Growth Inventory (PTGI) was used for the assessment of positive psychological changes after the experience of critical incidents [14, 33, 34]. The PTGI consists of five subscales with satisfying internal consistency (Cronbach α in brackets): “Relating to Others” (7 items, e.g. ‘I accept needing others’; *α* = .85), “New Possibilities” (5 items, e.g. ‘I developed new interests’; *α* = .84), “Personal Strength” (4 items, e.g. ‘I discovered that I’m stronger than I thought I was’; *α* = .72), “Spiritual Change” (2 items, e.g. ‘I have a stronger religious faith’; *α* = .85) and “Appreciation of Life” (3 items, e.g. ‘Appreciating each day’; *α* = .67) [34]. The items were rated on a 6-point Likert scale from 0 = “not at all” to 5 = “very strong” and subsequently a PTG total score was computed [3, 14, 35], which was only used in this study.

#### Sense of coherence scale 29 item version (SOC-29)

Sense of coherence was assessed by means of the 29-item German version of the Sense of Coherence Scale (SOC-29), which consists of three subscales with satisfying internal consistency (Cronbach α in brackets): Comprehensibility (11 items, e.g. ‘Do you have a feeling that you are in an unfamiliar situation and don’t know what to do?’ (from ‘very often’ to ‘very seldom or never’); *α* = .79), Manageability (10 items, e.g. ‘When something unpleasant happened in the past your tendency was’: (from ‘to eat yourself up about it’ to ‘to say “ok that’s that, I have to live with it” and “go on”); *α* = .81) and Meaningfulness (8 items, e.g. ‘Doing the things you do every day is’: (from ‘a source of deep pleasure and satisfaction’ to ‘it’s certain that something will happen to spoil the feeling’); *α* = .86). The items were rated on a 7-point Likert scale from 1 to 7, followed by the calculation of a SOC total score, which was only used in further statistical analyses [36–38].

### Statistical analyses

As a first step, Pearson’s correlation statistics were conducted in order to investigate the relationships between study variables. Next, a two-step cluster analysis was carried out using the PTG total score in addition to the IES-R-Subscales Intrusion, Avoidance and Hyperarousal for the measurement of PTSS as input variables to examine if the constructs of PTG and PTSS exist independently from one another. For this procedure, the SOC total score plus the PTSD-Probability were used as evaluation fields. Subsequently, Univariate Analyses of Variance (ANOVAs) were performed to investigate cluster differences in SOC, PTG and PTSS, whereas alpha-level was set to *p* < .01 to control for alpha inflation.

## Results

The final sample consisted of 266 ambulance service members (87 female); 216 participants were voluntary, unpaid members (81 female) while 50 participants were full-time, paid members (6 female). 198 were trained Emergency Medical Technicians (70 female) and 68 trained Critical Care Paramedics (17 female). Ages ranged from 18 to 73 years (*M* = 29.94; *SD* = 11.07) and years of service ranged from one to 52 years (*M* = 9.91; *SD* = 9.04). With regards to the highest completed education level, 17 participants had finished compulsory school, 76 had completed a professional apprenticeship, 113 had a high-school diploma and 60 had a university degree. Furthermore, 156 service members were involved in a relationship and 110 were single. Participants had regularly performed between one and 25 ambulance services per month (*M* = 6.12; *SD* = 5.76), where the duration of one ambulance service lasted from 6 to 48 h (*M* = 11.84; *SD* = 2.92).

### Differences between voluntary and full-time and male and female ambulance personnel

Additionally, we performed univariate Analyses of Variance (ANOVAs) to investigate whether there are differences between voluntary and full-time ambulance personnel in SOC, PTSS and PTG. ANOVAs showed significant differences in the PTSD-Probability (*F*_(1,265)_ = 7.67, *p* < .01) and in the PTSS symptom Hyperarousal (*F*_(1,265)_ = 8.98, *p* < .01) between full-time and voluntary ambulance personnel. In detail, voluntary ambulance personnel showed a lower PTSD-Probability (*M* = − 3.68, *SD* = .90) as well as a lower level of Hyperarousal (*M* = 2.69, *SD* = 4.61) than full-time ambulance personnel (PTSD-Probability: *M* = − 3.25, *SD* = 1.32; Hyperarousal: *M* = 5.06, *SD* = 6.59). In addition, no significant differences were found between full-time and voluntary ambulance personnel in SOC and PTG.

Moreover, we also conducted ANOVAs to investigate whether there are differences in SOC, PTSS and PTG between male and female ambulance personnel. ANOVAs revealed significant differences between male and female ambulance personnel in SOC (*F*_(1,265)_ = 4.49, *p* < .05), in the PTSD-Probability (*F*_(1,265)_ = 6.34, *p* < .05) plus in the PTSS symptoms Hyperarousal (*F*_(1,265)_ = 8.61, *p* < .01) and Intrusion (*F*_(1,265)_ = 12.21, *p* < .01). In detail, male ambulance personnel showed a higher level of SOC (*M* = 119.37, *SD* = 18.76) as well as lower levels in the PTSS-domains PTSD-Probability (*M* = − 3.71, *SD* = .92), Hyperarousal (*M* = 2.50, *SD* = 4.39) and Intrusion (*M* = 5.12, *SD* = 5.85) than female ambulance personnel (SOC: *M* = 113.71, *SD* = 23.50; PTSD-Probability: *M* = − 3.38, *SD* = 1.14; Hyperarousal: *M* = 4.44, *SD* = 6.18; Intrusion: *M* = 8.00, *SD* = 7.14). No significant differences were found between male and female ambulance personnel in PTG.

## Correlation analyses

Initial correlation analyses (see Table 1) for the investigation of relationships between PTSS, PTG and SOC revealed that higher levels of PTG were associated with higher levels of SOC (*r* = .27, *p* < .01). In particular, correlation analyses also showed that especially the SOC-subscale Meaningfulness was positively associated with the PTG Total Score (*r* = .27, *p* < .01) as well as with the PTG-subscales New Possibilities (*r* = .27, *p* < .01), Relating to Others (*r* = .31, *p* < .01) and Appreciation of Life (*r* = .14, *p* < .05). Furthermore, higher levels of SOC were associated with lower levels of the PTSS symptom Avoidance (*r* = −.17, *p* < .01) and with a lower PTSD-Probability (*r* = −.14, *p* < .05). No significant correlations were found between PTG and PTSS.

**Table 1:** Means, standard deviations, and intercorrelations of study variables

	M	SD	1	2	3	4	5	6
Measures								
1. PTG	43.38	15.03	–					
2. SOC	117.52	20.56	.27**	–				
IES-R (PTSS)								
3. Intrusion	6.06	6.43	.11	−.07	–			
4. Avoidance	5.83	6.44	.10	−.17**	.60**	–		
5. Hyperarousal	3.14	5.12	.04	−.09	.71**	.60**	–	
6. PTSD-Probability	−3.60	1.01	.06	−.14*	.68**	.83**	.94**	–

*Notes.* *p < .05, **p < .01

*PTG* = Post-Traumatic Growth Total Score, *SOC* = Sense of Coherence Total Score, *IES-R* = Impact of Event Scale Revised, *PTSS* = Post-Traumatic Stress Symptoms, *PTSD* = Post-Traumatic Stress Disorder

Furthermore, additional correlation analyses were conducted to investigate possible relationships between SOC, PTSS and PTG with years of service and duration of ambulance services. These analyses revealed that more years of service were positively associated with a higher level of SOC (*r* = .15, *p* < .05) as well as negatively associated with lower levels of the PTSS symptom Avoidance (*r* = −.14, *p* < .05) and a lower PTSD-Probability (*r* = −.13, *p* < .05). Beyond that, correlation analyses also showed that a lower duration of ambulance services was associated with higher levels of PTG (*r* = −.16, *p* < .01). No significant correlations were found between PTSS and duration of ambulance services as well as between PTG and years of service.

### Two-step cluster analysis

A two-step cluster analysis was then conducted to investigate whether PTG and PTSS existed independently from one another. PTG as well as the IES-R-Subscales Intrusion, Avoidance and Hyperarousal were used as input variables. As outliers have been reported to strongly influence the results of a cluster analysis [39], the data was investigated for possible outliers before the implementation of the cluster analysis. Fortunately, no outliers were discovered. Furthermore, satisfying internal consistencies were found for the total scores of PTG (*α* = .84) and SOC (*α* = .75) as well as for the IES-R-Subscales Intrusion (*α* = .85), Avoidance (*α* = .76) and Hyperarousal (*α* = .82).

In cluster analysis, four clusters were identified and labelled as followed: Cluster A (PTSS-low/PTG-low) included 66 participants who scored low on the Post-Traumatic Stress symptoms (PTSS: Intrusion, Avoidance, Hyperarousal) and low on the PTG total score. Cluster B (PTSS-low/PTG-high) included 107 participants who scored low on PTSS and high on the PTG total score. Cluster C (PTSS-high/PTG-high) included 55 participants who scored high on PTSS and high on the PTG total score. Lastly, Cluster D (PTSS-high/PTG-low) included 38 participants who scored high on PTSS and low on the PTG total score.

### Univariate analyses of variance

**Fig. 1:**
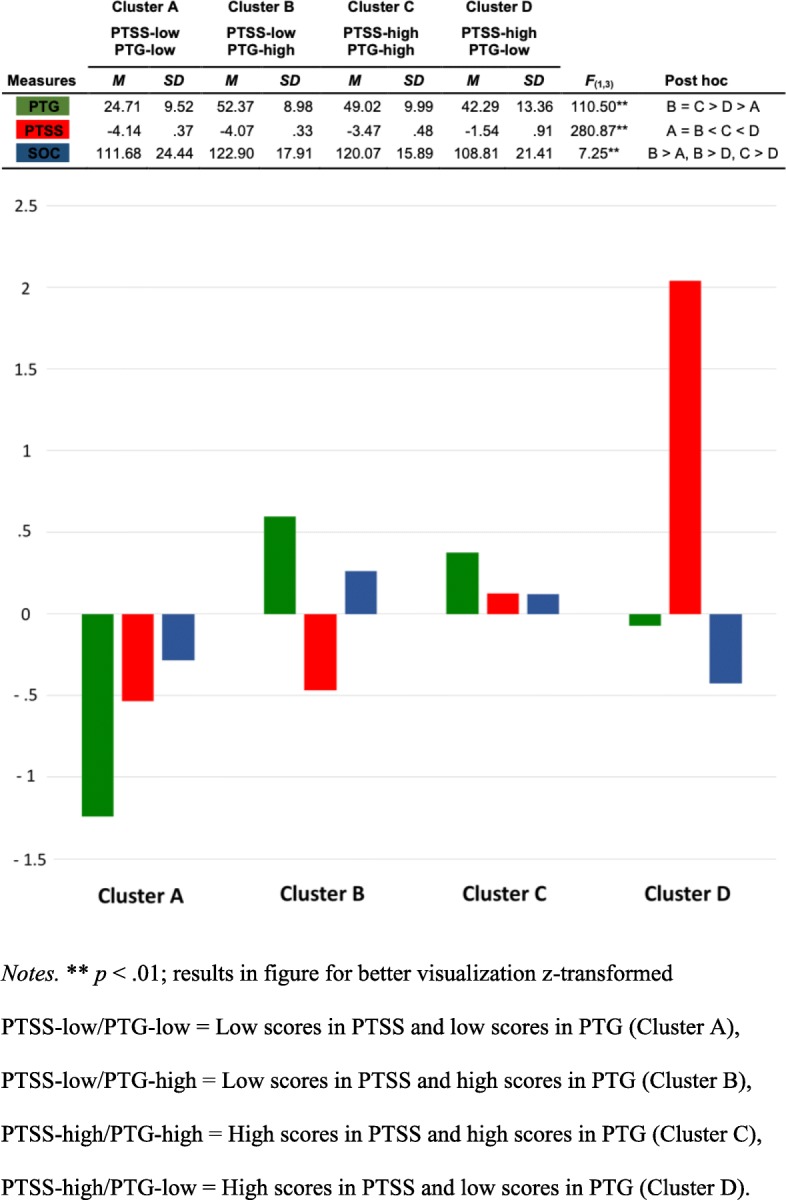
Cluster Differences (ANOVAs) in Post-Traumatic Growth (PTG), Post-Traumatic Stress Symptoms (PTSS) and Sense of Coherence (SOC)

Finally, we performed univariate Analyses of Variance (ANOVAs) on the defined four clusters to investigate whether SOC is linked to PTG despite the coexistence of PTSS. The total scores for PTG and SOC, the PTSS criteria Intrusion, Avoidance and Hyperarousal as well as the PTSD-Probability were considered as dependent variables (see Table 2 and for better visualization, Fig. 1).

**Table 2:** Cluster Differences (ANOVAs) in Post-Traumatic Growth (PTG), Post-Traumatic Stress Symptoms (PTSS; Intrusion, Avoidance, Hyperarousal), PTSD-Probability and Sense of Coherence (SOC)

	A		B		C		D				
	PTSS-low/PTG-low		PTSS-low/PTG-high		PTSS-high/PTG-high		PTSS-high/PTG-low				
	M	SD	M	SD	M	SD	M	SD	F_(1,3)_	eta^2^	Post hoc
Measures											
1. PTG	24.71	9.52	52.37	8.98	49.02	9.99	42.29	13.36	110.50**	.56	A < D < C = B*
IES-R											
2. Intrusion	3.03	3.27	2.50	2.28	10.13	3.42	15.50	8.74	116.29**	.57	B = A < C < D*
3. Avoidance	1.95	2.58	2.93	3.14	8.07	4.61	17.47	5.33	173.80**	.67	A = B < C < D*
4. Hyperarousal	.97	2.02	.87	1.39	3.55	2.81	12.68	6.79	145.55**	.63	B = A < C < D*
5. PTSD-Probability	−4.14	.37	−4.07	.33	−3.47	.48	−1.54	.91	280.87**	.76	A = B < C < D*
6. SOC	111.68	24.44	122.90	17.91	120.07	15.89	108.81	21.41	7.25**	.08	A < B, D < B, D < C*

*Notes.* *p < .05 **p < .01

*PTG* = Post-Traumatic Growth Total Score), *PTSS* = Post-Traumatic Stress symptoms (Intrusion, Avoidance, Hyperarousal), *PTSD* = Post-Traumatic Stress Disorder, *PTSS-low/PTG-low* = Low scores in PTSS and low scores in PTG (Cluster A), *PTSS-low/PTG-high* = Low scores in PTSS and high scores in PTG (Cluster B), *PTSS-high/PTG-high* = High scores in PTSS and high scores in PTG (Cluster C), *PTSS-high/PTG-low* = High scores in PTSS and low scores in PTG (Cluster D), *IES-R* = Impact of Event Scale Revised, *SOC* = Sense of Coherence Total Score)

#### Cluster differences in PTG

ANOVAs showed significant cluster differences in PTG (*F*_(1,3)_ = 110.50, *p* < .01): Clusters with PTG-high (Clusters B and C) did not differ regarding PTG and showed a significant, higher level of PTG than clusters with PTG-low (Cluster A and D). Furthermore, the cluster with PTSS-high (Cluster D) showed a higher level of PTG than the cluster with PTSS-low (Cluster A).

#### Cluster differences in PTSS

Regarding PTSS, ANOVAs showed significant cluster differences for all IES-R subscales (Intrusion: (*F*_(1,3)_ = 116.29, *p* < .01; Avoidance: *F*_(1,3)_ = 173.80, *p* < .01; Hyperarousal: *F*_(1,3)_ = 145.55, *p* < .01) as well as for the PTSD-Probability (*F*_(1,3)_ = 280.87, *p* < .01): Clusters with PTSS-low (Clusters A and B) did not differ regarding PTSS and showed a significant, lower level of PTSS than clusters with PTSS-high. Here, the cluster with PTG-high (Cluster C) showed a lower level of PTSS than the cluster with PTG-low (Cluster D).

#### Cluster differences in SOC

In addition, ANOVAs also showed significant cluster differences in SOC (*F*_(1,3)_ = 7.25, *p* < .01): The cluster with PTG-high and PTSS-low (Cluster B) showed a higher level of SOC than clusters with PTG-low (Clusters A and D). Furthermore, the cluster with PTG-high and PTSS-high (Cluster C) showed a higher SOC-level than the cluster with PTG-low and PTSS-high (Cluster D).

## Discussion

In this study, we tried to detect new possibilities to improve the working conditions for voluntary and full-time ambulance personnel. To that end, we examined the impact of SOC on PTSS and PTG in Austrian ambulance personnel. We hypothesized that PTG and PTSS were related to SOC but also existed independently from one another. Therefore, SOC might be linked to PTG despite the possible presence of PTSS.

Regarding the relationships between SOC, PTG and PTSS our findings concur with already existing literature in this particular field of research [12, 21, 22, 26, 27]: Higher levels of SOC are associated with higher levels of PTG as well as with lower levels in the PTSS-domains Avoidance and PTSD-Probability in Austrian ambulance personnel.

Additionally, we found indications that a higher level of SOC seems to increase the probability for the development of PTG in Austrian ambulance personnel after the experience of critical incidents in the line of duty. Notably, we found positive associations between the SOC-subscale Meaningfulness and the PTG Total Score (*r* = .27, *p* < .01) plus the PTG-subscales New Possibilities (*r* = .27, *p* < .01), Relating to Others (*r* = .31, *p* < .01) and Appreciation of Life (*r* = .14, *p* < .05). Maybe, those findings underline the relevance of SOC and the possible significant role of the SOC-subscale Meaningfulness for the development of PTG after experiencing a critical incident. Possibly, an explanation for this could be found through the assumption that people who are in general quite capable in finding more meaningfulness in their life (SOC-dimension) should therefore also be quite capable in finding more meaningfulness in a distressing life event [19–21]. In addition, this ability to find meaningfulness is assumed to grow while struggling with the distressing life event; this struggle is also seen as essential for the development of PTG later on in the adjustment process [13]. Therefore, ambulance personnel might be especially capable of finding meaningfulness in distressing life events because of the constant struggle with such events in the line of duty. Subsequently, this constant struggle could have a beneficial effect for the development of PTG later on. Concerning this, Linley and Joseph [40] found indications that an enhanced SOC seems to predict PTG in psychotherapists who are also often confronted with distressing events. Furthermore, a study by Triplett et al. [41] found a positive relationship between the existence of meaningfulness in a person’s life and PTG.

However, the majority of published studies so far regarding the relationship between SOC and PTG mostly highlight the role of the SOC-dimension meaningfulness for the development of PTG. Yet, some studies could be found, who concentrate on the SOC-construct as a whole or on the remaining SOC-dimensions comprehensibility or manageability. In this context, a study by Arya and Davidson [42] should be mentioned in particular, because they found indications for a positive association between the SOC-construct as a whole and the PTG total score. Furthermore, they also detected positive relationships between all the three SOC-dimensions comprehensibility, manageability and meaningfulness and the PTG-dimensions Relating to Others, Spiritual Change and the PTG total score. Moreover, Nishi et al. [27] found positive relationships between all three SOC-dimensions and the PTG-dimensions “Personal Strength” as well as “Relating to Others” in a sample of accident survivors. Furthermore, a study by Kennedy et al. [43] regarding patients in rehabilitation after a spinal cord injury, could prove a relationship between the SOC-dimension manageability and stress related growth. Furthermore, it should also be pointed out that some studies found indications for a negative relationship between SOC and PTG. Concerning this, a study by Brockhouse et al. [44] revealed a negative relationship between a lower SOC and a higher level of PTG. Also, there could be some studies found, which either reported a positive relationship between SOC and PTG or no significant relationship at all [40, 45]. On that account, future studies should concentrate their focus in particular on the relationships between the SOC- and PTG-dimensions for a more thorough and detailed examination of this topic.

Regarding ambulance personnel, a further possible reason for the found link between SOC and the development of PTG after critical incidents might be the already implemented peer support system in the Austrian Red Cross [46, 47]. More specifically, this peer support system consists of specially trained ambulance officers who provide psychological support for colleagues after they have experienced a distressing event in the line of duty. As a result, the supported ambulance personnel increases their ability to reduce their stress level and to regain their operational ability [24]. Furthermore, this peer support is likely to enhance the confidence of the supported ambulance officers to handle their job-related duties further on (SOC-dimension manageability) and to find a deeper meaning in the experience of the distressing incident (SOC-dimension meaningfulness) [19–21].

Concerning this, as previously mentioned, we found positive associations between the SOC-subscale Meaningfulness and the PTG-subscales New Possibilities, Relating to Others, Appreciation of Life plus the PTG Total Score. Perhaps, ambulance personnel might profit from an incorporation of this study results in the peer support system of the Austrian Red Cross. In detail, members of this support system could receive further training to promote SOC among their colleagues (e.g. to encourage their colleagues in finding a deeper meaning out of a distressing event which was experienced in the line of duty). As a consequence, the peers could become more competent in promoting the constructs of SOC and PTG among Austrian ambulance personnel, whereby their readiness for duty and the possibility for the development of PTG after experiencing a distressing incident might be increased. Supporting this, a study by Linley, Joseph and Loumidis [45] assumed, that the construct of SOC could promote positive adaption processes after traumatic experiences (e.g. PTG).

In contrast, it could also be possible that the implemented peer support system could have negative effects. In literature it is theorized, that the premature addressing of distressing experiences (e.g. during the course of a peer support session) could enhance the risk of experiencing a renewed loss of control over the distressing situation as well as the risk of experiencing an overflow through intrusive memories [48]. As a consequence, this overflow could enhance trauma related intrusions as well as avoidance behaviour and therefore, complicate the overcoming of distressing experiences [46].

The generally high level of SOC in Austrian ambulance personnel might also be attributed to the professional preparedness through their ongoing training, including expert knowledge regarding the management of medical emergencies. Acquiring additional psychological knowledge (e.g., through psychoeducation as part of the training) could also facilitate the ability to find meaningfulness in a distressing event. This theory is supported by Streb [12] and Langeland et al. [25] who found that ambulance personnel with professional training showed a higher SOC-level and a lower PTSS-level than non-prepared colleagues. Furthermore, the availability of psychological support also leads to higher SOC-levels and lower PTSS-levels [12, 25]. Further studies will be needed to determine whether an increase in professional training and psychological interventions might be able to further increase the SOC-level and lower the PTSS-level in Austrian ambulance personnel.

Compared to other professions, ambulance personnel might be generally more likely to develop PTG if confrontation with fundamental existential topics (e.g. the meaning of life or the possibility of afterlife) is an integral part of their daily duties. For instance, resuscitating a patient after a heart attack could possibly lead to more intense thoughts about their own mortality. Subsequently, this could be followed by the development of a deepened meaning of spirituality or an increased appreciation of life, all well known as dimensions of PTG [13, 16]. Concerning this, our sample showed similar PTG total scores (*M* = 43.38, *SD* = 15.03) than firefighters [2] (*M* = 42.75, *SD* = 22.12). However, our sample also showed higher PTG total scores when compared to people who did not work in these professions but had experienced traumatic events in their private lives – for example a sample of men with prostate cancer (*M* = 38.37, *SD* = 26.64) in a study by Walsh et al. [49] as well as a sample of head and neck cancer survivors (*M* = 30.80, *SD* = 19.70) in a study by Holtmaat et al. [50].

Additionally, the results of the cluster analysis included a cluster (Cluster C) which consisted of ambulance personnel who showed high PTSS-levels as well as high PTG-levels. This underlines the hypothesis of Tedeschi and Calhoun [13] that PTSS and PTG represent independent constructs which could be present at the same time as the memory of the event itself could remain distressingly separate from experience of growth [13, 15, 16]. Potentially, the combination of professional training and being confronted with extremely distressing events – including incidents with many dead or severely wounded people, a dead or severely wounded child or colleague, a strong personal identification with the victims, mortal danger and extreme interest of the media [24] – is particularly likely to elicit both PTG and PTSS.

However, further studies are required to investigate possible differences in developing PTG after experiencing a distressing event in the line of duty in addition to the potential beneficial effects of SOC on PTG in voluntary and full-time Austrian ambulance personnel. Up to this point, it also remains unclear whether there are differences between voluntary and full-time ambulance personnel in the extent of regular confrontation with distressing events and in the regeneration from it.

Moreover, the results of this study can only be interpreted with simultaneous consideration of potential limitations. Since the current study concentrated on Austrian ambulance personnel the results might not be comparable to studies from other countries with different concepts of professional education and training. For example, a study by Pohl-Meuthen et al. [51] showed significant differences among European ambulance services regarding organization, infrastructure, occupational qualification, duration of training and the level of medical qualification. However, the consideration of voluntary ambulance personnel can be seen as a strength of the present study because, as far as we know, no other studies have addressed PTSS and PTG in voluntary ambulance personnel. Nonetheless, volunteers play a crucial role in the effective functioning of the Austrian emergency services (ambulance and fire service). In addition, while positive psychological changes after traumatic incidents (e.g. PTG) have become an important factor for the professional treatment of post-traumatic stress disorders, there is still some debate on the definition and measurement of PTG in research [16]: Previous insights on this subject rest upon the results of cross-sectional studies, mostly using the PTGI [14] as a retrospective measurement of self-reported growth after traumatic incidents. Thus, the participants are instructed to recall their inner mental state prior to a traumatic event in retrospect and subsequently evaluate the extent of positive change after the event happened [16]. This could be quite a challenging psychological task which may never have happened spontaneously on its own. Indeed, this circumstance could have thoroughly influenced the given PTGI answers substantially [16]. Furthermore, regarding the symptoms of Post-Traumatic Stress (PTSS), it should be noted that in our current study no PTSD diagnosis were assigned. While the IES-R [29, 30] is a reliable tool for the self-assessment of the three major DSM-IV-TR criteria for Post-Traumatic Stress - Intrusion, Avoidance and Hyperarousal [7, 31, 32] – it is not sufficient for diagnosing PTSD. Moreover, the present study found a significant, though small, correlation between SOC and PTG (*r* = .27; *p* < .01) which could be an indication for a possible link between SOC and PTG. However, because this study was designed as a cross-sectional study and did not compare SOC with another traits, it is not justified to say that our findings point to SOC playing an important role in the development of PTG after the experience of critical incidents. For this purpose, it is necessary for future studies to use a longitudinal study design to examine possible facilitating influences of SOC on PTG.

## Conclusions

Our results indicate a significant association between SOC and the development of PTG after critical incidents in Austrian ambulance personnel. Additionally, PTG and PTSS appear to be independent from each other and can consequently be prevalent at the same time. However, more research is needed on the mechanisms leading to PTG and preventing PTSS in voluntary vs. full-time ambulance personnel.

## Abbreviations

ANOVA: Univariate Analysis of Variance
DSM-V: Diagnostic and Statistical Manual of Mental Disorders - Fifth Edition
IES-R: Impact of Event Scale Revised
PTG: Post-Traumatic Growth
PTGI: Post-Traumatic Growth Inventory
PTSD: Post-Traumatic Stress Disorder
PTSS: Post-Traumatic Stress Symptoms
SOC: Sense of Coherence
SOC-29: Sense of Coherence Scale 29 Item Version
